# Machine-learning models to predict myopia in children and adolescents

**DOI:** 10.3389/fmed.2024.1482788

**Published:** 2024-11-19

**Authors:** Jingfeng Mu, Haoxi Zhong, Mingjie Jiang

**Affiliations:** Shenzhen Eye Hospital, Shenzhen, China

**Keywords:** machine learning, myopia, influencing factors, children and adolescents, predictive model

## Abstract

**Objectives:**

To explore machine-learning applications in myopia prediction and analyze the influencing factors of myopia.

**Methods:**

Stratified cluster random sampling was used to select elementary school students in Shenzhen, China for inclusion in this case-control study. Myopia screening, ocular biological parameter measurements, and questionnaires were conducted. Random forest (RF), decision tree (DT), extreme gradient boosting trees (XGBoost), support vector machine (SVM), and logistic regression (LR) algorithms were used to construct five myopia prediction models using R software (version 4.3.0). These myopia prediction models were used to investigate the relationship between ocular biological parameters, environmental factors, behavioral factors, genetic factors, and myopia.

**Results:**

This study included 2,947 elementary school students, with a myopia prevalence rate of 47.2%. All five prediction models had an area under the receiver operating characteristic curve (AUC) above 0.75, with prediction accuracy and precision exceeding 0.70. The AUCs in the testing set were 0.846, 0.837, 0.833, and 0.815 for SVM, LR, RF, and XGBoost, respectively, indicating their superior predictive performance to that of DT (0.791). In the RF model, the five most important variables were axial length, age, sex, maternal myopia, and feeding pattern. LR identified axial length was the most significant risk factor for myopia [odds ratio (OR) =8.203], followed by sex (OR = 2.349), maternal myopia (OR = 1.437), Reading and writing posture (OR = 1.270), infant feeding pattern (OR = 1.207), and age (OR = 1.168); corneal radius (OR = 0.034) and anterior chamber depth (OR = 0.516) served as protective factors.

**Conclusion:**

Myopia prediction models based on machine learning demonstrated favorable predictive performance and accurately identified myopia risk factors, and may therefore aid in the implementation of myopia prevention and control measures among high-risk individuals.

## Introduction

Myopia is a global public health concern (1) that affected 1.4 billion individuals worldwide in 2020 with a prevalence of 22.9%. It has been projected that this number will increase to 4.7 billion individuals by 2050, resulting in a prevalence of 49.8% (2). In 2020, 52.7% of children and adolescents in China were myopic (3), which contributed to the position of China as the country with the highest number of individuals with myopia (4). Myopia often develops during childhood and adolescence, and myopia development during this period is more likely to progress to high myopia, increasing the risk of various eye diseases such as glaucoma, cataracts, macular degeneration, and retinal detachment. High or pathological myopia significantly increases the risk of blindness (5) and is a major cause of blindness (6). Myopia imposes an economic burden on society (7). In Singapore, an average of $148 is spent on myopia-related costs per adolescent with myopia per year (8). Globally, uncorrected refractive errors result in an estimated annual economic loss of approximately $202 billion (9).

Currently, the etiology of myopia remains unclear, although its development in children and adolescents is thought to be influenced by a combination of environmental, behavioral, and genetic factors (10). Myopia has been associated with sex (10), age (10), near work (11, 12), time spent outdoors (10), duration of electronic device use (10), sleep (13), and parental myopia (14). Exploring influencing factors of myopia occurrence and development and conducting myopia risk assessments are of significant importance for improving the ocular health of children and adolescents. Owing to potential interactions among various influencing factors, such as the co-occurrence of increased screen time, long periods of near work, and reduced time spent outdoors, conventional statistical methods may fail to identify covariance and potential confounding factors (15). Therefore, novel analytical methods are required to mitigate the influence of confounding factors, identify covariates, and measure the magnitude and significance of interactions between variables.

Continuous advancements in computer technology have increased the use of artificial intelligence (AI) in many fields of medicine, including ophthalmology (16). The application of AI in the study of ophthalmic diseases has evolved from its initial focus on diabetic retinopathy (17), age-related macular degeneration (18), and glaucoma (19) to encompass anterior segment disorders such as refractive errors (20). Machine learning, a core component of AI, is increasingly used in the diagnosis, treatment, and prognostic assessment of various diseases (21) and has recently been applied to myopia research, including in the prediction of axial length and identification of influencing factors (21–25). However, the use of machine learning for the prediction of myopia risk remains relatively rare. Previous myopia prediction studies based on machine learning have often failed to comprehensively include feature variables and targeted indicators (26). This study therefore constructed myopia prediction models using multiple indicators, including behavioral habits, dietary habits, genetic factors, and ocular biological parameters, and five machine-learning algorithms: random forest (RF), decision tree (DT), extreme gradient boosting trees (XGBoost), support vector machine (SVM), and logistic regression (LR). The aims of the study were to explore machine-learning applications in myopia prediction, analyze the factors influencing myopia, and provide scientific evidence for targeted myopia prevention, control measures, and policy recommendations.

## Methods

### Sample size

Sample size was estimated in accordance with the methodology outlined by Riley et al. (27) using the pmsampsize() function in R software (The R Foundation for Statistical Computing, Vienna, Austria). This calculation determined that a minimum of 2,060 study participants were required, with the non-response rate set at 20%.

### Study participants

A stratified cluster sampling method was used to select the study population of 2,947 primary school students (744, 528, 527, 360, 459, and 329 in grades 1, 2, 3, 4, 5, and 6, respectively) from Shenzhen in June 2022. The study was approved by the Ethics Committee of Shenzhen Eye Hospital (Approval No. 20201230-06), and informed consent was obtained from the legal guardians of all the participants.

The inclusion criteria were as follows: (1) current enrollment in a primary school in Shenzhen, (2) ability to cooperate with ophthalmic examinations, and (3) guardians able to complete questionnaires. The exclusion criteria were as follows: (1) presence of organic eye diseases, and (2) inability to complete ophthalmic examinations.

### Data collection

#### Myopia screening

Myopia screening was performed using visual acuity tests and dioptric detection, and conducted by ophthalmologists following the Chinese Health Standard (WS/T 663-2020) (28). Visual acuity was assessed using visual acuity charts, and non-cycloplegic refraction was performed using an autorefractometer. Autorefraction was performed three times per eye, and the average value was calculated to obtain a reliable refractive error reading for each participant.

#### Ocular biometric measurements

The ocular biometric parameters of the study participants were measured using an optical biometer. The measured parameters included axial length, central corneal thickness, corneal radius, anterior chamber depth, and lens thickness.

#### Questionnaire

A questionnaire was developed to include the following content: (1) sociodemographic characteristics, including sex, age, height, and weight; (2) parental myopia status; (3) dietary habits, including the frequency of consumption of sugary foods, sugary beverages, fried foods, vegetables, and fruits; and (4) behavioral factors, including reading and writing posture, duration of continuous reading and writing, sleep duration, and time spent outdoors. Teachers distributed the questionnaires to the guardians of the study participants, who completed them online.

The questionnaire included items assessing adherence to the traditional Chinese guideline known as “yi quan, yi chi, yi cun,” which translates to “one fist, one foot, one inch.” This principle relates to specific distances that should be maintained during reading and writing to promote proper posture and reduce the risk of myopia among schoolchildren. One fist: a distance of one fist (approximately 10 cm) is advised between the chest of a student and the edge of their desk. One foot: a distance of one foot (approximately 33 cm) should be maintained between the eyes of the student and their reading material. One inch: pens or pencils should be held at a distance of one inch (approximately 3.3 cm) from the tip.

#### Diagnostic criteria for myopia

Participants with an uncorrected visual acuity <5.0 and spherical equivalent refraction < −0.50 diopter and Participants those wore orthokeratology lenses were defined as myopic (28). Myopia was classified based on the spherical equivalent refraction of the right eye. Mild myopia is defined as −3.00 diopter ≤spherical equivalent refraction <−0.50 diopter, moderate myopia is defined as −6.00 diopter ≤spherical equivalent refraction <−3.00 diopter, and high myopia is defined as spherical equivalent refraction <−6.00 diopter.

### Construction and evaluation of prediction models

#### Feature variables

Multivariate logistic regression was used to identify the statistically significant influencing factors of myopia, namely feature variables.

#### Data splitting

Stratified random sampling (according to myopia status) was used to classify the 2,947 study participants into training (2,064 students) and testing sets (883 students) at a ratio of 7:3.

#### Data modeling

**Figure 1:**
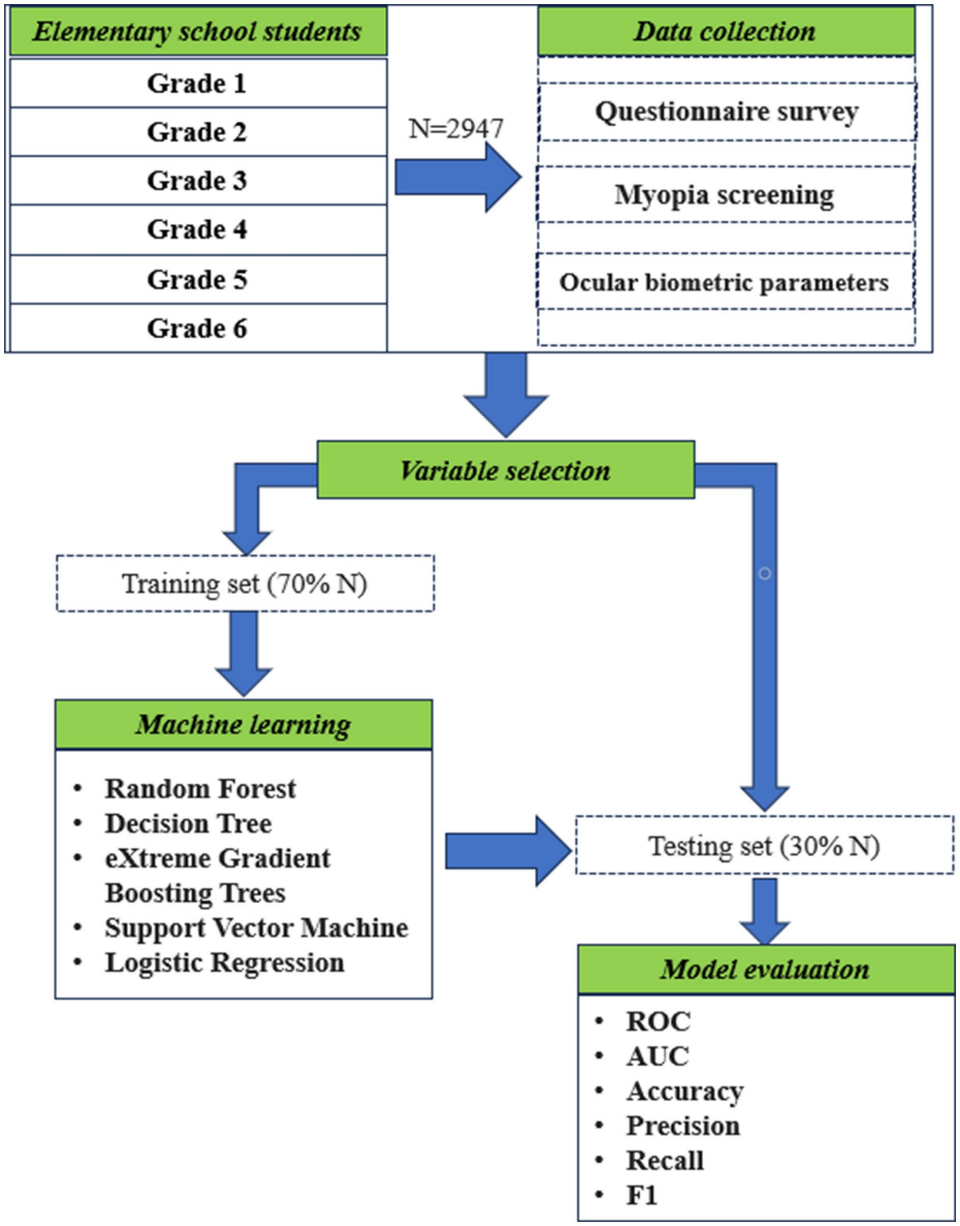
Flow chart of the study.

Feature variables were incorporated into the machine-learning model. The five machine-learning algorithms RF, DT, XGBoost, SVM, and LR were used to construct myopia prediction models from the training data set. The machine-learning modeling process is illustrated in Figure 1.

#### Model evaluation

The testing set was used to determine model performance metrics such as accuracy, precision, recall, and *F*_1_-score. The receiver operating characteristic (ROC) curves were plotted and used to calculate the area under the curve (AUC) and 95% confidence interval. The DeLong test (29) was conducted to assess and compare the performance of the models.

#### Analysis of feature variable importance

The importance of independent variables reflects their relationship with the dependent variable and explains their contribution to the predictive performance of the model. Mean decrease accuracy and odds ratios (ORs) were used to assess the importance of feature variables to the prediction models. The OR represents the odds of an event in group 1 compared to those in group 2, where odds means the event over non-event.

### Statistical analysis

Machine-learning prediction models were constructed, and statistical analyses were performed using R software (version 4.3.0). $\bar{x}\pm s$ was used to describe for normally distributed continuous data; intergroup comparisons were performed using independent samples *t*-tests. Non-normally distributed continuous data are expressed as P_50_ (P_25_, P_75_), and intergroup comparisons were conducted using the Wilcoxon rank-sum test. Count data are presented as frequencies, and intergroup comparisons were performed using the chi-squared test. The significance level (*α*) for all tests was set at 0.05, with *p* < 0.05 indicating statistical significance.

## Results

### Basic information

**Figure 2:**
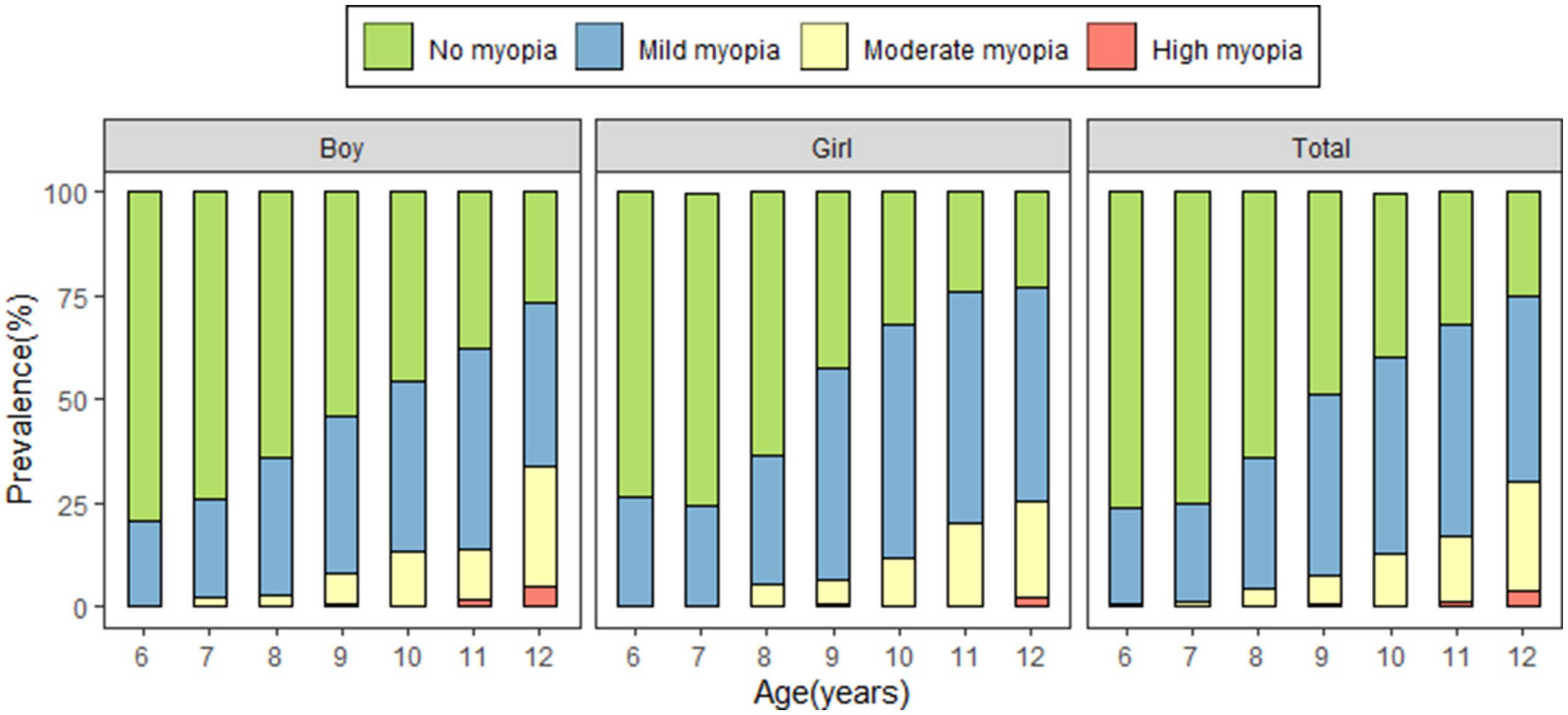
Prevalence of myopia among participants.

The study included 2,947 primary school students (1,617 boys and 1,330 girls). The minimum age of the participants was 6 years, and their maximum age was 12 years, with a 50th percentile of 9 years. The prevalence of myopia among the participants was 47.2%. The prevalence of myopia increased with age, and was 24.1, 33.3, 52.6, 55.6, 67.8, and 75.7% in grades 1, 2, 3, 4, 5, and 6, respectively (*χ*^2^ = 436.475, *p* < 0.001). The prevalence of myopia was significantly higher in girls (50.5%) than in boys (44.5%) (*χ*^2^ = 10.539, *p* < 0.001). Myopia became more severe with age, with the prevalence of moderate myopia increasing significantly from 0.7% at the age of 6 years to 26.3% at the age of 12 years (Figure 2).

### Feature variables

**Figure 3:**
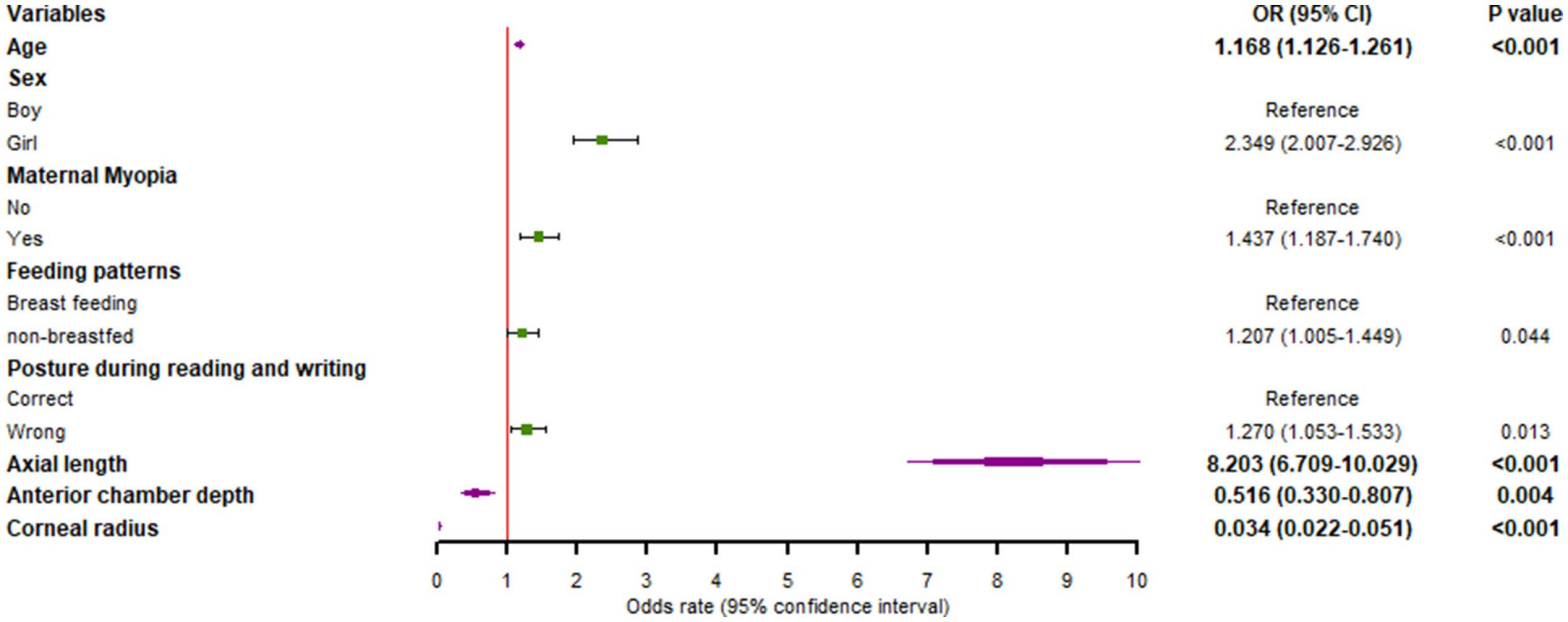
Odds ratios of feature variables in the myopia prediction model based on logistic regression.

Logistic regression analysis identified eight statistically significant influencing factors of myopia: age (OR = 1.168), sex (OR = 2.349), maternal myopia (OR = 1.437), infant feeding pattern (OR = 1.207), reading and writing posture (OR = 1.270), axial length (OR = 8.203), anterior chamber depth (OR = 0.516), and corneal radius (OR = 0.034) (Figure 3).

### Comparison of training and testing sets

**Figure 4:**
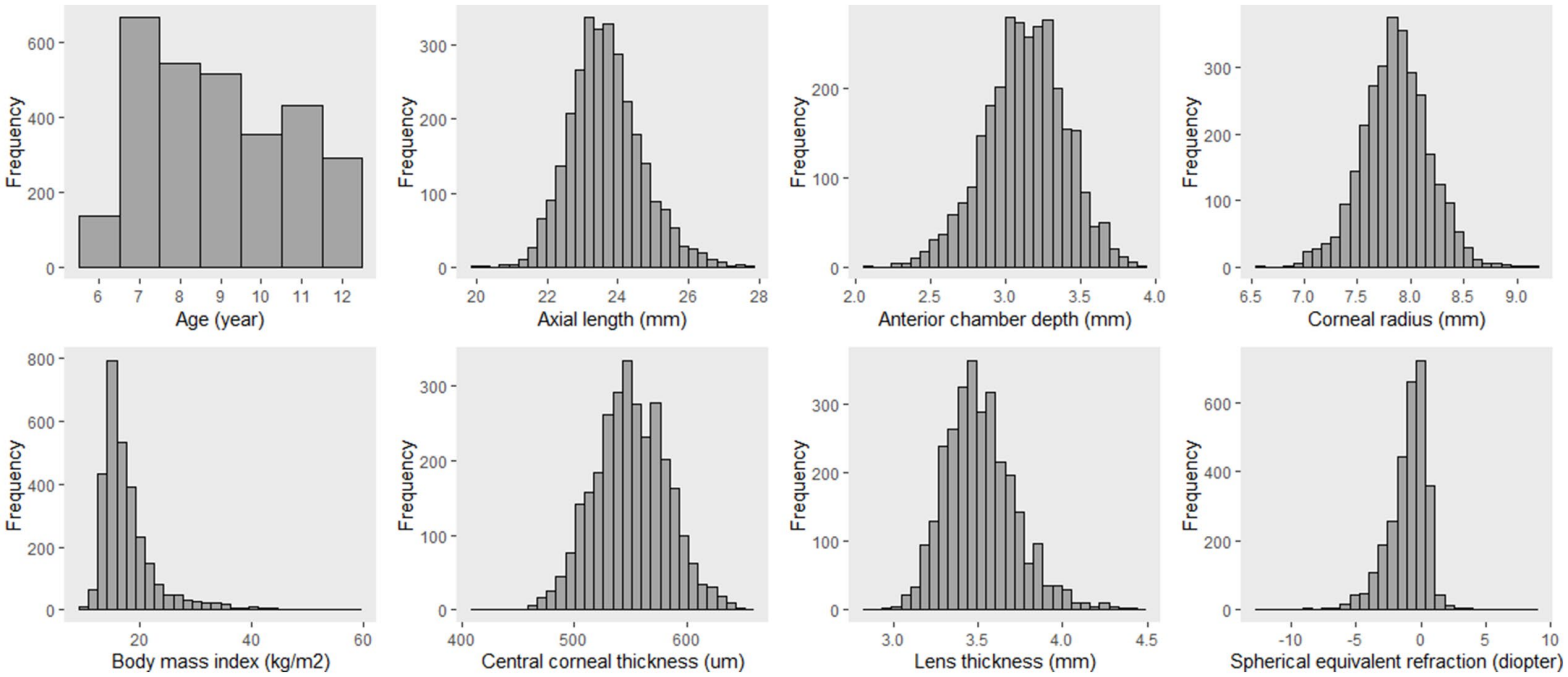
Distribution of quantitative data in the study.

The quantitative data were not normally distributed and are presented as P_50_ (P_25_, P_75_) (Figure 4). The prevalence of myopia and feature variables did not differ significantly between the training and testing sets (*p* < 0.05), indicating a balanced allocation between the two datasets (Table 1).

**Table 1:** Comparison of myopia and feature variables between training and testing sets.

Variables	Training set	Testing set	Statistical value	*p*
Myopia (*n*)				
No	1,089	466	*χ*^2^ = 0.000	0.995
Yes	975	417		
SE (D)	−0.625 (−1.625, 0)	−0.625 (−1.75, 0)	*z* = 1.099	0.272
Age [P_50_ (P_25_, P_75_), years]	9 (7, 10)	9 (7, 11)	*z* = −0.814	0.416
Body mass index [P_50_ (P_25_, P_75_), kg/m^2^]	16.5 (14.9, 19.2)	16.6 (14.8, 19.2)	*z* = −0.677	0.499
Axial length [P_50_ (P_25_, P_75_), mm]	23.58 (22.95, 24.28)	23.61 (23.01, 24.29)	*z* = −1.240	0.215
Central corneal thickness [P_50_ (P_25_, P_75_), μm]	549.00 (527.00, 572.00)	552.00 (531.00, 574.00)	*z* = −1.988	0.050
Anterior chamber depth [P_50_ (P_25_, P_75_), mm]	3.14 (2.96, 3.31)	3.14 (2.95, 3.33)	*z* = 0.109	0.913
Lens thickness [P_50_ (P_25_, P_75_), mm]	3.50 (3.37, 3.63)	3.49 (3.36, 3.63)	*z* = 0.214	0.830
Corneal radius [P_50_ (P_25_, P_75_), mm]	7.85 (7.66, 8.06)	7.87 (7.66, 8.07)	*z* = *−*0.333	0.739
Sex (*n*)				
Boy	1,122	495	*χ*^2^ = 0.720	0.396
Girl	942	388		
Paternal myopia (*n*)				
No	1,374	598	*χ*^2^ = 0.372	0.542
Yes	690	285		
Maternal myopia (*n*)				
No	1,245	559	*χ*^2^ = 2.324	0.127
Yes	819	324		
Birth (*n*)				
Eutocia	1,139	499	*χ*^2^ = 0.442	0.506
Caesarean	925	384		
Infant feeding pattern (*n*)				
Breast feeding	1,057	470	*χ*^2^ = 1.007	0.316
Non-breastfed	1,007	413		
Sugared beverages intake (*n*)				
<3 time/week	318	151	*χ*^2^ = 1.326	0.250
≥3 time/week	1,746	732		
Sweet food intake (*n*)				
<3 time/week	93	47	*χ*^2^ = 0.912	0.340
≥3 time/week	1,971	836		
Fried food intake (*n*)				
<3 time/week	259	109	*χ*^2^ = 0.024	0.878
≥3 time/week	1,805	774		
Fruit intake (*n*)				
≥3 time/week	1,415	621	*χ*^2^ = 0.909	0.340
<3 time/week	649	262		
Vegetable intake (*n*)				
≥3 time/week	1,460	642	*χ*^2^ = 1.174	0.279
<3 time/week	604	241		
Sleep duration (*n*)				
≥9 h	920	391	*χ*^2^ = 0.021	0.884
<9 h	1,144	492		
Reading and writing posture (*n*)				
Correct	788	343	*χ*^2^ = 0.116	0.733
Wrong	1,276	540		
Posture of holding pen (*n*)				
Correct	1,318	571	*χ*^2^ = 0.176	0.675
Wrong	746	312		
*Per capita* monthly income (*n*)				
≥10,000 yuan	988	405	*χ*^2^ = 0.994	0.319
<10,000 yuan	1,076	478		
Living area per person (*n*)				
≥20 m^2^	1,258	567	*χ*^2^ = 2.793	0.095
<20 m^2^	806	316		
Time spent outdoors per day (*n*)				
≥2 h	757	346	*χ*^2^ = 1.661	0.197
<2 h	1,307	537		

### Evaluation of prediction models

**Figure 5:**
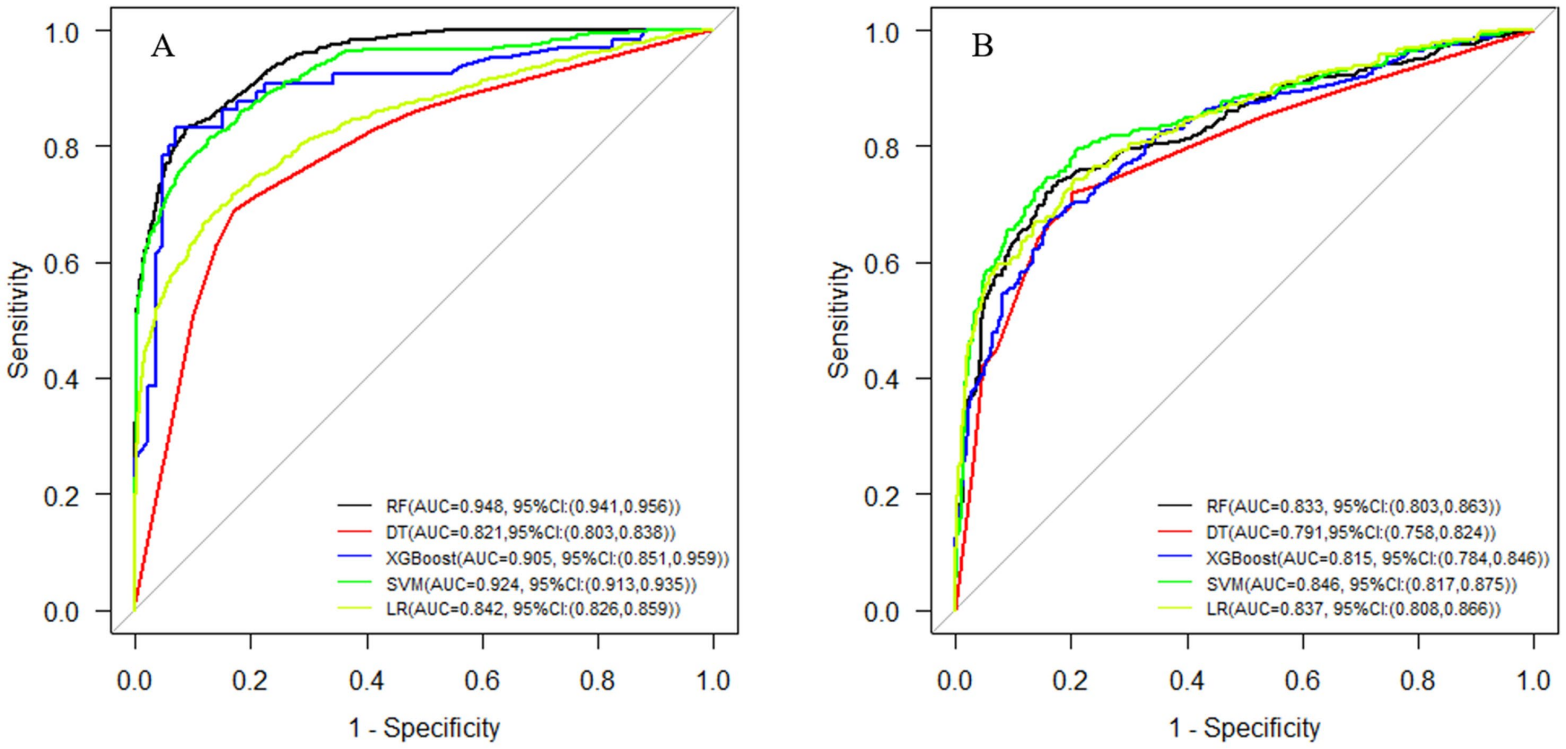
ROC curves of different machine-learning models. (A) Training set. (B) Testing set. ROC, receiver operating characteristic.

The RF, DT, XGBoost, SVM, and LR prediction models were constructed using the training set and evaluated using testing set. The AUCs of the ROC curves were greater than 0.8 for all the models except the DT. Among the models, the SVM exhibited the highest accuracy, precision, recall, and *F*_1_-score, indicating a superior predictive performance for myopia (Table 2 and Figure 5).

**Table 2:** Evaluation of predictive performance of models based on the testing set.

Models	AUC (95% CI)	Accuracy	Precision	Recall	*F*_1_
Random forest	0.833 (0.803, 0.838)	0.764	0.738	0.776	0.756
Decision tree	0.791 (0.758, 0.824)	0.757	0.785	0.670	0.723
eXtreme gradient boosting trees	0.815 (0.784, 0.846)	0.754	0.765	0.693	0.727
Support vector machine	0.846 (0.817, 0.875)	0.844	0.851	0.812	0.831
Logistic regression	0.837 (0.808, 0.866)	0.783	0.830	0.678	0.746

AUC, the area under the curve.

### Importance of feature variables

**Figure 6:**
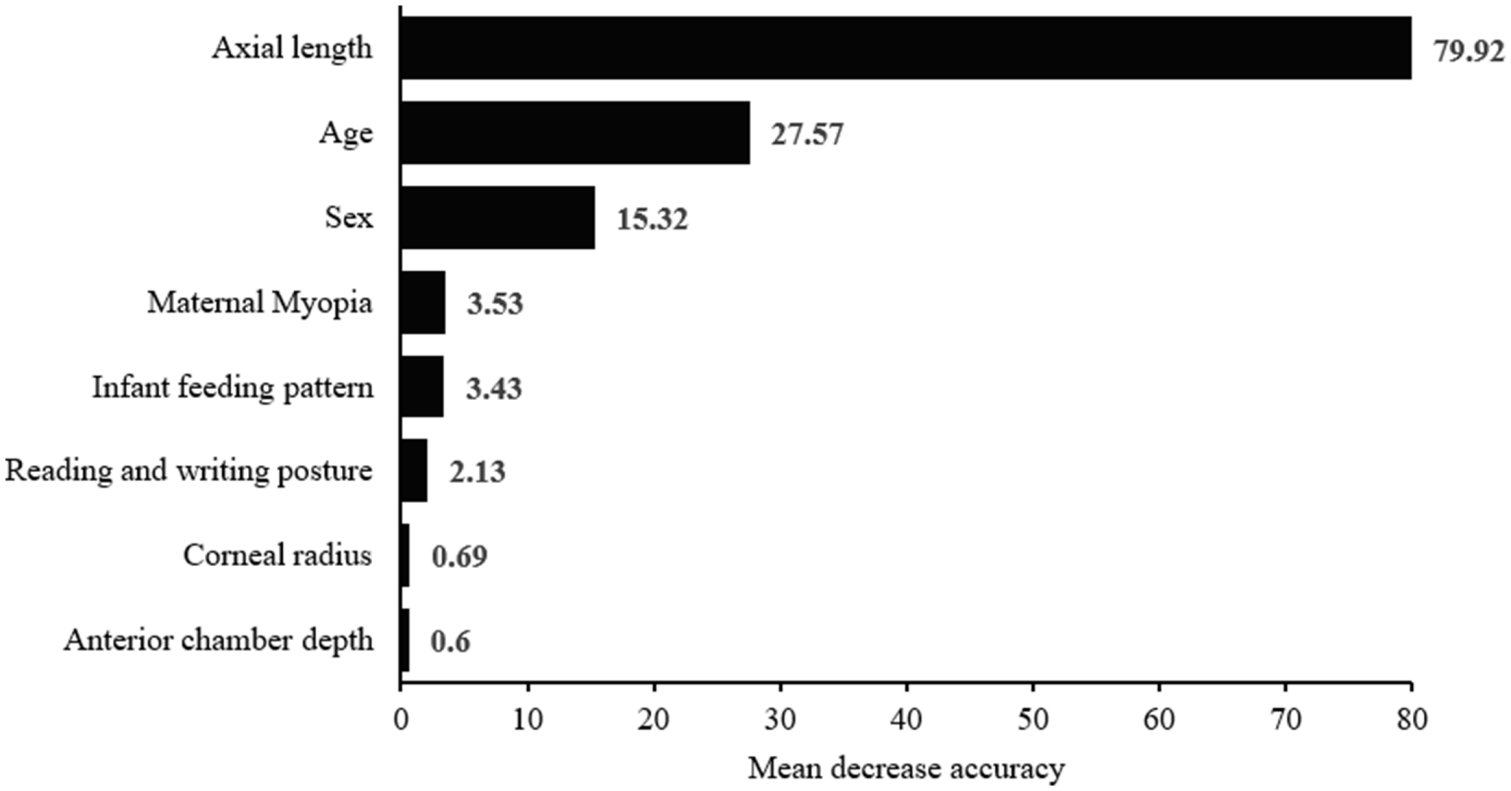
Importance of feature variables for the prediction model constructed using the random forest algorithm.

As shown in Figure 5, the optimal prediction model in the training set was RF, whereas in the testing set the optimal myopia prediction model was SVM, followed by LR. Analysis of variable importance in the RF model revealed axial length, age, sex, maternal myopia, and infant feeding pattern to be the five most important variables, as shown in Figure 6.

## Discussion

In this study, the prevalence of myopia among 2,947 primary school students was 47.2%, with rates increasing with age. Five machine-learning algorithms were used to construct myopia risk prediction models that all achieved AUC values over 0.75 and accuracy and precision values over 0.7. The AUCs for prediction models created using the SVM, LR, RF, and XGBoost algorithms were 0.846, 0.837, 0.833, and 0.815, respectively, indicating superior predictive performance compared to that of the prediction models generated using the DT (AUC, 0.791) and XGBoost (AUC, 0.815) algorithms.

The machine-learning models in this study outperformed those constructed in previous studies. A study of primary school students in Jiamusi, China reported AUCs of 0.710, 0.606, 0.682, and 0.620 for RF, DT, XGBoost, and SVM models, respectively (30). A study of primary and secondary school students in Chengdu, China reported AUCs of 0.768, 0.767, and 0.769 for LR, XGBoost, and SVM models, respectively (26). One possible reason for this difference is that the previous studies involved a relatively narrow selection of feature variables, with one relying solely on questionnaires to collect information on participant behaviors (26, 30). The present study collected addition information on the ocular biometric parameters closely associated with myopia.

Analysis of the importance of feature variables in myopia prediction models in the present study and revealed a close association between ocular biometric parameters and myopia. The refractive status of the eye has been shown to be determined by the lens, axial length, and corneal radius (31), with axial length and corneal curvature being the most crucial factors affecting ocular refraction in children and adolescents (32, 33). This is consistent with the findings of the present study, which showed that axial length, anterior chamber depth, and corneal radius were the principal biological parameters influencing myopia. Refractive errors result from a mismatch between the imaging focal length and the axial length. The active mechanism of emmetropization involves axial elongation, whereas the passive mechanism primarily involves the regulation of corneal and lens diopters (34). Axial length gradually increases with age, with a 1-mm increase in axial length corresponding to a myopic shift of 0.69D in spherical equivalent refraction (34). Studies have identified that the axial length/corneal radius ratio as an important parameter for determining myopia in children and adolescents, significantly superior to axial length alone (34). However, the axial length/corneal radius ratio threshold for myopia determination varies among different populations, with reported optimal thresholds of 2.906 for Australian children and adolescents (35) and 3.0 for Singaporean children and adolescents (36). Changes in anterior chamber depth are inversely proportional to lens thickness, with studies indicating that, anterior chamber depth increases by up to 0.18 mm due to lens deformation and displacement following ciliary muscle paralysis (34).

Studies have reported associations between dietary habits (37), sleep (38), near work (39), parental myopia (39), and time spent outdoors (40) and the occurrence and progression of myopia. The present study assessed the influence of environmental and behavioral factors on myopia through questionnaires, and identified sex as a significant factor, with girls at a greater risk of myopia than boys (41). Consistent with this, the probability of myopia in East Asian females has been shown to be twice that in age-matched males, although studies in South Asian and Latin American populations did not find sex to be a significant factor (42). The relationship between myopia and sex may be complicated by factors such as education, time spent outdoors, and economic conditions. The present study also identified parental myopia, specifically maternal myopia, as a significant influencing factor of myopia. Whole-genome studies have associated more than 150 nucleotide polymorphisms with myopia (43).

In the present study, an incorrect reading and writing posture was found to increase the risk of myopia. This is consistent with previous research involving children aged 9–11 years, which showed that children who read and wrote at a distance of less than 30 cm had higher degrees of myopia and greater progression of myopia over a 6-month period (44). This may be the result of a compensatory mechanism, whereby children with existing myopia hold reading materials closer to see more clearly, further exacerbating myopia. The association between breastfeeding and myopia has been receiving increasing attention. The present study identified infant feeding pattern as an influencing factor of myopia. A previous study of children aged 6–12 years found that myopia prevalence was lower among breastfed children (51.8%) than among non-breastfed children (64.7%) (45). Aksoy et al. (46) demonstrated that in first- and second-grade elementary school students, non-breastfed children were more likely to have refractive errors than were children who were exclusively or partially breastfed. The present study additionally found that body mass index (BMI) influenced myopia in the RF model. Large-scale population studies have shown a J-shaped relationship between BMI and myopia, with both low and high BMI associated with myopia (47).

The present study has some limitations. First, the diagnosis of myopia was based on non-cycloplegic refraction. Non-cycloplegic refraction has been shown to have high sensitivity and specificity in myopia screening among students (48), and myopia prevalence may have been overestimated to some extent. Second, the use of a questionnaire introduces the possibility of recall bias. Finally, the case-control design of the study limited its ability to determine causal relationships. Future prospective cohort studies are required to further elucidate the etiology of myopia using machine-learning algorithms.

In conclusion, the present study identified axial elongation, female sex, thickening of the lens, maternal myopia, incorrect reading and writing posture, and increased age as risk factors for myopia; corneal radius was a protective factor against myopia. Machine-learning models constructed using these factors accurately predicted myopia and may be used to identify high-risk populations. Cohort studies will be conducted to validate the identified myopia risk factors and elucidate their causal relationships.

## Data Availability

The original contributions presented in the study are included in the article/supplementary material, further inquiries can be directed to the corresponding author.
